# The Anti-Tumor Activity of Succinyl Macrolactin A Is Mediated through the β-Catenin Destruction Complex via the Suppression of Tankyrase and PI3K/Akt

**DOI:** 10.1371/journal.pone.0141753

**Published:** 2015-11-06

**Authors:** Sushil C. Regmi, Su Young Park, Seung Joo Kim, Suhrid Banskota, Sajita Shah, Dong-Hee Kim, Jung-Ae Kim

**Affiliations:** 1 College of Pharmacy, Yeungnam University, Gyeongsan, Republic of Korea; 2 Research and Development Center, Daewoo Pharm. Co. Ltd, Busan, Republic of Korea; Taipei Medicine University, TAIWAN

## Abstract

Accumulated gene mutations in cancer suggest that multi-targeted suppression of affected signaling networks is a promising strategy for cancer treatment. In the present study, we report that 7-*O*-succinyl macrolactin A (SMA) suppresses tumor growth by stabilizing the β-catenin destruction complex, which was achieved through inhibition of regulatory components associated with the complex. SMA significantly reduced the activities of PI3K/Akt, which corresponded with a decrease in GSK3β phosphorylation, an increase in β-catenin phosphorylation, and a reduction in nuclear β-catenin content in HT29 human colon cancer cells. At the same time, the activity of tankyrase, which inhibits the β-catenin destruction complex by destabilizing the axin level, was suppressed by SMA. Despite the low potency of SMA against tankyrase activity (IC_50_ of 50.1 μM and 15.5 μM for tankyrase 1 and 2, respectively) compared to XAV939 (IC_50_ of 11 nM for tankyrase 1), a selective and potent tankyrase inhibitor, SMA had strong inhibitory effects on β-catenin-dependent TCF/LEF1 transcriptional activity (IC_50_ of 39.8 nM), which were similar to that of XAV939 (IC_50_ of 28.1 nM). In addition to suppressing the colony forming ability of colon cancer cells *in vitro*, SMA significantly inhibited tumor growth in CT26 syngenic and HT29 xenograft mouse tumor models. Furthermore, treating mice with SMA in combination with 5-FU in a colon cancer xenograft model or with cisplatin in an A549 lung cancer xenograft model resulted in greater anti-tumor activity than did treatment with the drugs alone. In the xenograft tumor tissues, SMA dose-dependently inhibited nuclear β-catenin along with reductions in GSK3β phosphorylation and increases in axin levels. These results suggest that SMA is a possible candidate as an effective anti-cancer agent alone or in combination with cytotoxic chemotherapeutic drugs, such as 5-FU and cisplatin, and that the mode of action for SMA involves stabilization of the β-catenin destruction complex through inhibition of tankyrase and the PI3K/Akt signaling pathway.

## Introduction

Constitutive activation of PI3K signaling is one of the most frequent gene mutations in human cancer [1,2] and results in the activation of the downstream signaling molecule Akt, which signals cell proliferation and survival. In colorectal cancer (CRC), *PIK3CA*, which encodes PI3K catalytic alpha polypeptide, is frequently amplified or mutated. Because the mutated PIK3CA plays an important role in cancer cell survival, proliferation, and metabolism, *PIK3CA* is suggested as a therapeutic target in CRC [3]. Additionally, inappropriate activation of the Wnt pathway has also been observed in CRC as well as many other cancers [4–6]. This Wnt pathway activation results in the nuclear translocation of β-catenin and the modulation of the T-cell factor/lymphoid enhancer factor (TCF/LEF) transcriptional activity to promote cell proliferation [7]. The two signaling pathways of PI3K/Akt and Wnt/β-catenin cross-talk through glycogen synthase kinase 3β (GSK3β). GSK3β is a component of the β-catenin destruction complex, which also includes axin, adenomatous polyposis coli (APC), and casein kinase 1. In the absence of Wnt, β-catenin is phosphorylated by GSK3β and undergoes proteosomal degradation. In the presence of Wnt, the activated Frizzled receptor with Dishevelled disrupts assembly of the β-catenin destruction complex, and β-catenin is stabilized in the cytosol [8]. Additionally, phosphorylation of GSK3β by PI3K/Akt in the presence of growth factors results in the inhibition of GSK3β kinase activity [9–11], which leads to stabilization of β-catenin. Therefore, β-catenin phosphorylation and degradation via GSK3β activation constitute one of the anti-cancer action mechanisms of PI3K/Akt inhibitors. In addition, axin, by acting as a β-catenin scaffold protein, regulates the efficiency of the β-catenin destruction complex [12]. GSK3β is stably bound to axin, which is the least abundant core component of the β-catenin destruction complex [12–14]. Furthermore, GSK3β binding of axin directs axin to target β-catenin and shields GSK3β from the action of Akt [15]. The axin levels are regulated by tankyrase, which induces axin ubiquitinylation and proteosomal degradation. Studies with XAV939, a selective and potent tankyrase inhibitor, have demonstrated that it stabilizes axin levels and promotes β-catenin destruction [16,17]. It has also been reported tankyrase 1 is overexpressed in a variety of cancers [18–20], and XAV939 has been shown to be an effective anti-cancer agent for CRC and other cancers [21]. Furthermore, the inhibition of the Wnt/β-catenin pathway by tankyrase inhibitors enhances the efficacy of EGFR inhibitor for the treatment of lung cancer [22], which suggests that tankyrase inhibitors could be used as adjuvant cancer treatments.

Macrolides have numerous biological activities, including the modulation of inflammation [23] and of the proliferation of B16-F10 murine melanoma cells [24]. In a previous study, we reported that the 7-*O*-succinyl derivative of macrolactin A (SMA) inhibits class I PI3K enzyme activity and its downstream signaling pathway, PI3K/Akt/mTOR/p70S6k, *in vitro* and *in vivo* [25]. In the present study, we report that SMA inhibits tankyrase in addition to inhibiting the PI3K/Akt pathway. Moreover, the study demonstrates that this dual inhibitory effect of SMA effectively suppresses the nuclear β-catenin level and TCF/LEF transcriptional activity *in vitro* and in a mouse xenograft tumor model.

## Materials and Methods

### Materials

Fetal bovine serum (FBS) and penicillin-streptomycin were purchased from Gibco (Grand Island, NY, USA). XAV939, 5-fluorouracil, and 3-(4,5-dimethylthiazol-2-yl)-2,5-diphenyltetrazolium bromide (MTT) were purchased from Sigma-Aldrich (St. Louis, MO, USA). BIO was purchased from Tocris Bioscience (Ellisville, MO, USA). Antibodies directed against phospho-p85/PI3K (Tyr 467), p85/PI3K, Akt, phospho-mTOR, mTOR, phospho-β-catenin (S33/37/T41), Axin1, and Axin2 were obtained from Cell Signaling Technology (Boston, MA). Recombinant human Wnt-3α was purchased from R&D Systems, Inc. (Minneapolis, MN). Akt, phospho-GSK-3β (S9), GSK-3β, c-Myc, cyclinD1, lamin B, and β-actin antibodies were from Santa Cruz Biotechnology (Santa Cruz, CA, USA). The β-catenin antibody was purchased from Sigma-Aldrich (St. Louis, MO). SMA was generated from *Bacillus polyfermenticus* KJS-2 by Daewoo Pharmaceutical (Busan, Korea). They provided SMA in a salt form that was soluble in PBS.

### Cell culture

The human colon epithelial cancer cell lines (HT29 and HCT116), the mouse colon carcinoma cell line CT26, and the human lung adenocarcinoma epithelial cell line A549 were purchased from the American Type Culture Collection (ATCC, VA, USA). HT29 cells were grown in RPMI1640 (Hyclone), whereas the CT26, A549, and HCT116 cell lines were grown in DMEM (Hyclone). Media was supplemented with 10% FBS containing 100 IU/ml penicillin and 100 μg/ml streptomycin. The cells were incubated at 37°C in a tissue culture incubator containing a 5% CO_2_/95% air atmosphere. After reaching confluence, the cells were trypsinized and sub-cultured by splitting the cells at a ratio of 1:5.

### Proliferation assay

Cells were seeded in a 96-well plate at 5000 cells/well, starved by culturing in 1% FBS overnight, and treated with SMA with or without 10% FBS, BIO (0.1 μM), or LiCl (10 mM) for 48 h. MTT solution (20 μl) was added, and the cells were incubated for 4 h at 37°C. The MTT solution was then discarded by aspiration and 200 μl of DMSO was added to dissolve formazan crystals. After 30 minutes of incubation, absorbance was measured at 540 nm using a microplate reader (Molecular Devices, Menlo Park, CA). Relative proliferations are expressed as the percentages of the proliferation of the vehicle-treated controls.

### Colony forming assay

Colony formation is an important endpoint for determining cancer cell proliferation and tumor responses to drugs. In the present study, colony formation was measured using an *in vitro* clonogenic assay. Agar growth medium solution (0.5%) was prepared by mixing 1% agar with 2 × RPMI1640 medium at a ratio of 1:1. Agar growth medium (0.5%; 500 μl) was added slowly to each 6-well plate. When sufficient colonies were visible and had reached a diameter *ca*. 0.5 mm, they were treated with the indicated concentrations of drugs. Media and drugs were changed every two days for 30 days. Colonies were stained with 0.005% crystal violet for 1 hour at 4°C, and the numbers of colonies were counted using a microscope connected to a digital camera (TMS; Nikon, Japan).

### Western blot analysis

Cells (CT26 and HT29) from 4- to 5-day-old cultures were seeded in a 60φ dish at 1×10^5^ cells/cm^2^. The cells were serum starved (1% FBS) overnight, and then treated with SMA for 1 h prior to stimulation with 10% FBS for 24 h. Total cytosolic and nuclear proteins were extracted from cells and tissues and subjected to a western blot as previously described [26].

### Nuclear translocation of β-catenin

The cells were grown on confocal dishes (glass bottomed dishes; Ibidi). Overnight serum starved cells were pretreated with SMA, stimulated with Wnt3α (10 ng/mL) or serum for 3 h, washed, fixed with 3.7% paraformaldehyde, permeabilized with 0.1% Triton X-100, blocked for 1 h with 3% bovine serum albumin (BSA) to reduce nonspecific binding, incubated with β-catenin antibody (1:100, Sigma) overnight at 4°C, and then incubated with a secondary antibody-conjugated with FITC (1:200) for 1 h at room temperature. Nuclei were stained with 600 nM 4,6-diamidino-2-phenylindole (DAPI), and then the cells were mounted with Prolong Gold Antifade (Invitrogen, Carlsbad, CA, USA). The cells were observed and photographed using a Confocal A1 Imaging System (Nikon, Japan). Between each of the steps, the cells were washed with Tris Buffered Saline containing 0.1% Tween 20 (TBST).

### TCF/LEF reporter dual-luciferase assay

The cells were grown on confocal dishes (glass bottomed dishes; Ibidi). Overnight serum starved cells were pretreated with SMA, stimulated with Wnt3α (10 ng/mL) or serum for 3 h, washed, fixed with 3.7% paraformaldehyde, permeabilized with 0.1% Triton X-100, blocked for 1 h with 3% bovine serum albumin (BSA) to reduce nonspecific binding, incubated with β-catenin antibody (1:100, Sigma) overnight at 4°C, and then incubated with a secondary antibody-conjugated with FITC (1:200) for 1 h at room temperature. Nuclear staining was conducted using 600 nM 4,6-diamidino-2-phenylindole (DAPI), and the cells were mounted with Prolong Gold Antifade (Invitrogen, Carlsbad, CA, USA). The cells were observed and photographed using an A1 Confocal Laser Microscope System (Nikon Corp., Tokyo, Japan). Between each step, the cells were washed with Tris Buffered Saline containing 0.1% Tween 20 (TBST).

### Tankyrase activity assay

To determine the effect of SMA on tankyrase 1 activity, the Tankyrase 1 Colorimetric Activity Assay Kit (Trivegen # 4700-096-K) was used. SMA was diluted in 1X I-PAR assay buffer (PAB). Histones from a histone-coated natural strip well plate were rehydrated with 50 μl of PAB and incubated at 25°C for 30 min. The PAB was removed and 25 μl of the assay cocktail, which consisted of 15 μl of the assay substrate and 10 μl of PAB with or without the inhibitor, was added to the samples. Tankyrase 1 enzyme (25 μl; 5 mUnits/well) was added to each well, and the tankyrase activities were determined using the Tankyrase 1 Colorimetric Activity Assay Kit according to the manufacturer's instructions.

The effect of SMA on Tankyrase 2 activity was screened using TNKS2 Histone Ribosylation Colorimetric Assay Kit (Bioscience #80583, San Diego, CA). The assay was performed as per the manufacturer’s instructions. Briefly, the transparent 96 well plate was coated with the histone proteins and incubated overnight at 4°C. A master mixture containing 10X PARP assay buffer, 10X PARP assay mixture and distilled water was prepared and added in all the wells except in substrate control where 10X PARP assay mixture was substituted by distilled water. 5 μl of SMA diluted in PBS were added to the designated wells at the indicated concentration. Then, the biotinylated NAD+ substrate was incubated with an assay buffer containing the TNKS2 enzyme. Finally, the streptavidin-HRP was added followed by the the colorimetric HRP substrate. The reaction was then stopped by the addition of 2M H_2_SO_4_. The produced color was then measured at 450 nm using Spectrostar Nano microplate reader (BMG LABTECH).

### Anti-tumor activity measurements in mouse tumor models

Six-week-old BALB/c mice were purchased from Orient Co. Ltd, Korea and housed under pathogen-free conditions in a 12-h light/dark cycle with access to food pellets and tap water *ad libitum*. The anti-tumor activities of MA and SMA were evaluated using an allograft (CT26 mouse colon carcinoma cell line) model and xenograft (HT29 human colon cancer and A549 human lung cancer cell line) models. Six mice per each group were used. In the allograft mouse model, the mice were injected subcutaneously with 5×10^6^ CT26 cells in 200 μl of DMEM/matrigel (1:1) into the flank region. Tumor sizes were measured every two days and calculated using following formula:

$$\text{V}\;({\text{mm}}^{3})=\text{L}\;\times \;\text{W}\;\times \;\text{W}\;\times \;½$$

When tumors reached 100 mm^3^, the mice were divided into different groups and treatments were started by injecting drugs i.p. daily. Mice were monitored for physical conditions such as body weight change and rustling behavior every day. When one mouse in 5-FU-treated group died after 7 days of 5-FU (50 mg/kg) treatment in the allograft tumor study, we ended this experiment, which was earlier than we planned. At the end of the study, the mice were sacrificed by placing them in a CO_2_ gas-filled chamber, and the excised tumors were measured and weighed. The xenograft models involved the same procedure except that HT29 or A549 cells were injected. Different doses of SMA, cisplatin, and of SMA + cisplatin were injected daily for 7 days with a 3-day break for a total of 28 days.

Mouse experiments were performed according to the institutional guidelines of the Institute of Laboratory Animal Resources (1996) and Yeungnam University for the care and use of laboratory animals (2009). The guidelines also contain a protocol for early euthanasia/humane endpoints for mice which become severely ill or die prior to the experimental endpoint. When mice became severely ill by losing 20% of body weight due to drug treatment or by gaining 20% of body weight due to tumor growth, mice were euthanized early, marking the endpoint of the experiment. This animal care and use protocol was reviewed and approved by the Institutional Animal Care and Use Committee at Yeungnam University (Approval number: 2014–020).

### Statistical analysis

Statistical analyses were performed using GraphPad Prism 5.0 software (San Diego, CA, USA). The results are expressed as means ± SEMs. A one-way analysis of variance with Tukey’s test was used for multiple comparisons between groups. Values of p < 0.05 were considered statistically significant.

## Results

### Dual inhibition of PI3K/Akt and Wnt/β-catenin signaling by SMA in colon cancer cells

**Fig 1:**
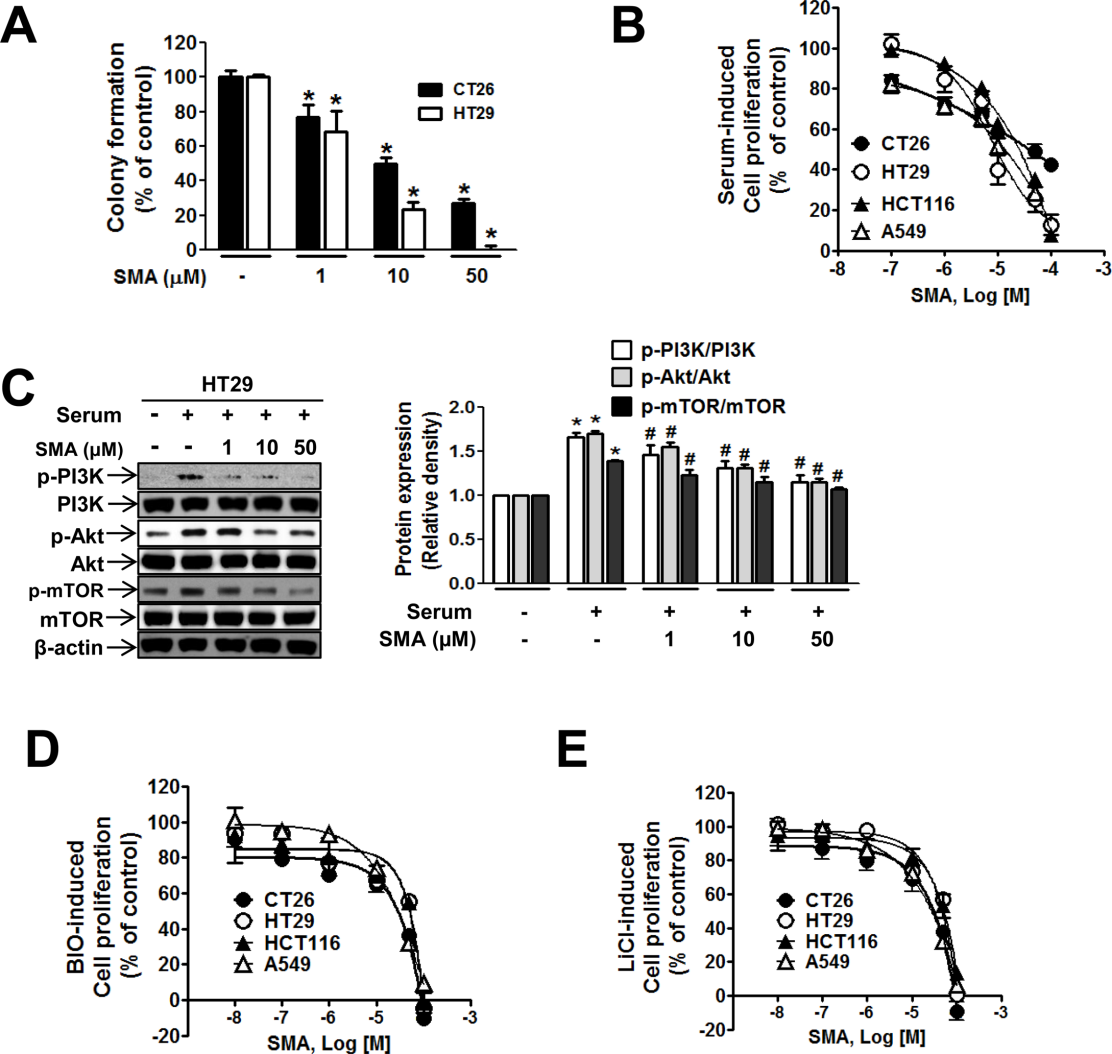
SMA suppressed colony formation and PI3K/Akt signaling in colon cancer cells. (A) Single-cell suspensions of CT26 and HT29 cells in agarose medium in the absence or presence of different concentrations of SMA were incubated for three weeks. Colonies that formed on five separate dishes were counted, and the mean colony numbers are shown in the bar graphs. ^*^ *P*<0.05 vs. the vehicle-treated control. (B) SMA potently inhibited cancer cell proliferation induced by serum. (C) Western blot analyses of the phosphorylation of PI3K/p85, Akt, and mTOR in SMA-treated HT29 colon cancer cells. The bar graph represents the relative band intensities of PI3K and its downstream signals. The measurements were performed in three independent experiments. ^*^ *P*<0.05 vs. the vehicle-treated control. ^#^ *P*<0.05 vs. serum-stimulated cells. D-E, GSK3β inhibitors (LiCl, BIO) induced cancer cell proliferation, which was inhibited by SMA.

The hyperproliferation of colonic epithelium is one of the earliest changes observed during the tumorigenesis of CRC [27,28]. We investigated whether SMA suppresses colon cancer proliferation by using a colony formation assay in HT29 cells, which possess an *APC* mutation, and CT26 cells, which do not. SMA significantly inhibited colony formation by colon cancer cells in a concentration-dependent manner (Fig 1A). In the cell proliferation assay, the IC_50_ (50% maximal inhibitory concentration) of SMA was lower in CT26 cells than in HT29 cells (Table 1). However, the inhibitory effect of SMA at concentrations above 10 μM was greater in HT29 cells than in CT26 cells (Fig 1B). In HT29 colon cancer cells, as we previously reported in endothelial cells, phosphorylation of PI3K and its downstream signals, Akt and mTOR phosphorylation, were blocked by SMA (Fig 1C). However, 50 μM SMA did not suppress Akt phosphorylation completely to the vehicle-treated control level. Because SMA at the same concentration completely suppressed colony formation, the results imply that PI3K/Akt signaling was not the only pathway mediating the inhibitory action of SMA against colon cancer cell proliferation. To identify other possible signaling pathways mediating the anti-proliferative action of SMA, we examined the involvement of β-catenin, which is associated with CRC tumorigenesis, in SMA action. The β-catenin level is regulated by GSK3β, which is inhibited by PI3K/Akt. Treatment with GSK3β inhibitors, BIO or LiCl, under serum-starved conditions, induced the proliferations of colon cancer cells, CT26, HT29, and HCT116. These cell proliferations were suppressed by SMA (Fig 1D and 1E). The IC_50_ of SMA in BIO- or LiCl-induced cell proliferation was higher than in serum-induced proliferation (Table 1).

**Table 1:** The half maximal inhibitory concentration (IC_50_) of SMA in cancer cell proliferation.

	IC_50_ (μM)			
	CT26 (WT APC, WT β-catenin)	HT29 (MT APC, WT β-catenin)	HCT116 (WT APC, MT β-catenin)	A549 (MT APC, WT β-catenin)
Serum-induced	18.2	30.2	25.1	13.5
BIO-induced	28.8	50.4	52.4	28.8
LiCl-induced	30.9	50.1	50.1	28.1

**Fig 2:**
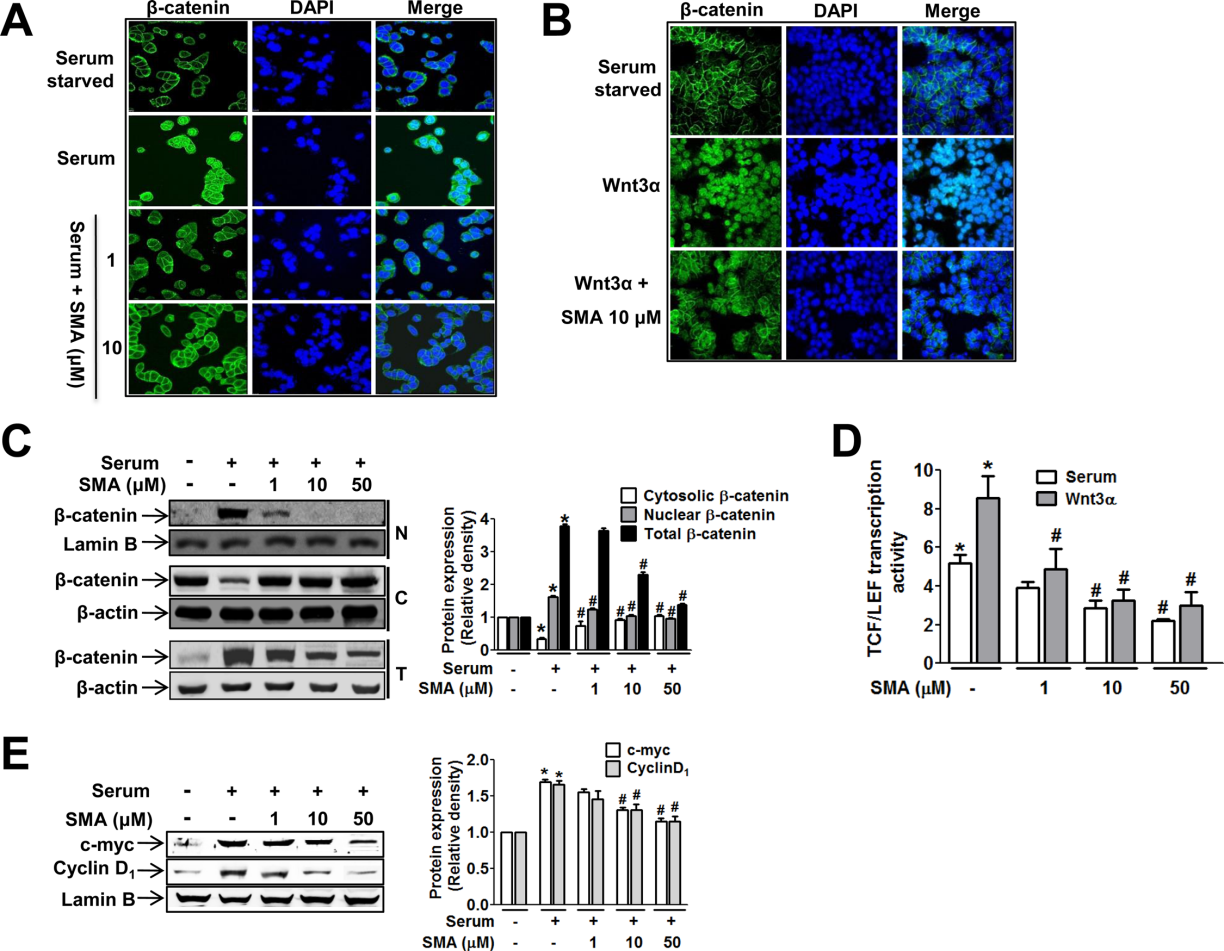
SMA inhibited nuclear translocation of β-catenin and TCF/LEF1 transcriptional activity in HT29 human colon cancer cells. (A-B) Immunofluorescence confocal images of β-catenin in HT29 cells treated with serum (A) or Wnt3α (B). β-Catenin and cell nuclei are stained green and blue, respectively. The images are representative of three independent experiments. (C) Western blots showing the levels of β-catenin in the cytosolic, nuclear, and total fractions of HT29 cells. The bar graph shows the relative band intensities of β-catenin. (D) HT29 cells were transfected with TCF/LEF-1-Luc reporter gene and pretreated with vehicle or SMA 1 hour prior to treatment with serum or Wnt3. ^*^ *P*<0.05 vs. vehicle-treated control cells. ^#^ *P*<0.05 vs. serum- or Wnt3α-treated cells. (E) The nuclear protein expressions of cyclin D1 and c-myc were inhibited in HT29 cells by SMA in a concentration-dependent manner. ^*^ *P*<0.05 vs. vehicle-treated control cells. ^#^ *P*<0.05 vs. serum-treated cells (n = 3).

Next, we found that SMA alters nuclear β-catenin level. Immunofluorescence staining with anti-β-catenin antibody and confocal microscopy demonstrated that cytosolic β-catenin was highly translocated to the nucleus after stimulation with serum (Fig 2A) or Wnt3α (Fig 2B) in HT29 cells. This nuclear translocation was significantly suppressed by SMA. In addition, Western blot analysis also demonstrated that serum-induced nuclear β-catenin accumulation was significantly suppressed by SMA. In fact, nuclear β-catenin was not detectable at SMA concentrations of 10 or 50 μM (Fig 2C). In addition, total cellular β-catenin levels were decreased by SMA. Given that nuclear β-catenin binds to members of the TCF/LEF1 family of transcriptional factors to induce target gene expressions, we examined the effects of SMA on TCF/LEF1 transcriptional activity using a TCF/LEF reporter gene dual-luciferase assay. Treating reporter gene-transfected cells with SMA significantly inhibited Wnt3α (10 ng/mL)- or serum-enhanced TCF/LEF transcription activities (Fig 2D). Furthermore, Western blot analysis of β-catenin/TCF/LEF target gene expressions showed that SMA significantly suppressed the expressions of cyclin D1 and c-myc in HT29 cells (Fig 2E).

### Effect of SMA on tankyrase enzyme activity, axin levels, and β-catenin destruction complex

**Fig 3:**
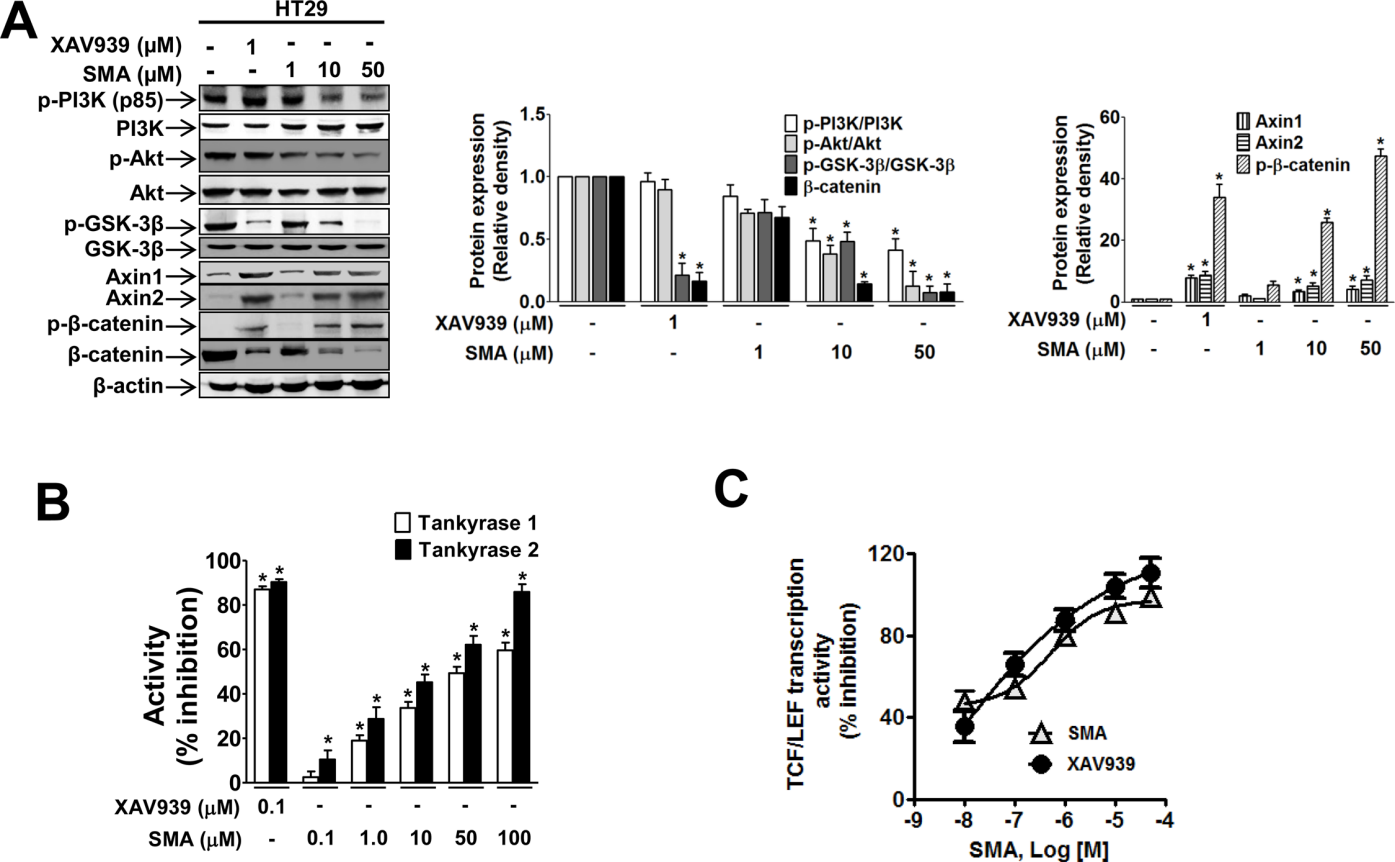
SMA facilitated GSK3β activity by inhibiting PI3K/Akt and tankyrase in HT29 colon cancer cells. (A) Western blot analyses of phosphorylation of PI3K, Akt, GSK3β, and β-catenin and the expression level of axin in SMA- or XAV939-treated HT29 cells. ^*^ *P*<0.05 vs. vehicle-treated controls. (B) Tankyrase activity was measured using the Tankyrase 1 Colorimetric Activity Assay Kit and TNKS2 Histone Ribosylation Colorimetric Assay Kit. XAV939 was used as a positive control. ^*^ *P*<0.05 vs. vehicle-treated control cells. (C) HT29 cells transfected with TCF/LEF-1-Luc reporter gene were pretreated with vehicle or SMA 1 hour prior to being treated with serum.

The SMA-induced decrease in total β-catenin levels suggests that SMA enhances the activity of the β-catenin destruction complex. In fact, SMA increased the level of the phosphorylated form of β-catenin (Fig 3A). This increase in β-catenin phosphorylation in SMA-treated cells may have been achieved by increased GSK3β activity due to suppression of PI3K/Akt activity or by increased levels of tankyrase-regulated axin proteins. As expected, there was an inhibitory effect of SMA on PI3K/Akt, and SMA suppressed GSK3β phosphorylation in a concentration-dependent manner (Fig 3A). At the same time, SMA increased axin1 and axin2 levels (Fig 3A). Because axin levels are regulated by tankyrase, we also investigated whether increased axin levels in SMA-treated cells were due to tankyrase inhibition by comparing the effect of SMA to the effect of XAV939, which is a selective and potent tankyrase inhibitor. SMA showed a low level of inhibition of tankyrase enzyme activity (IC_50_ of 50.1 μM and 15.5 μM for tankyrase 1 and 2, respectively) compared to XAV939 (Fig 3B and Table 2). However, SMA strongly inhibited TCF/LEF transcription activity to almost the same extent as XAV939 (Fig 3C). The IC_50_ values of SMA and XAV939 on TCF/LEF transcription activity were 39.8 nM and 28.1 nM, respectively (Table 2).

**Table 2:** The IC_50_ of SMA with respect to tankyrase and TCF/LEF transcription activities.

	IC_50_		
	Tankyrase 1 activity	Tankyrase 2 activity	TCF/LEF transcription activity
SMA	50.1 μM	15.5 μM	39.8 nM
XAV939	11 nM*	4 nM*	28.1 nM

*Previously reported [17].

### SMA inhibited tumor growth in syngenic and xenograft mouse colon cancer models

**Fig 4:**
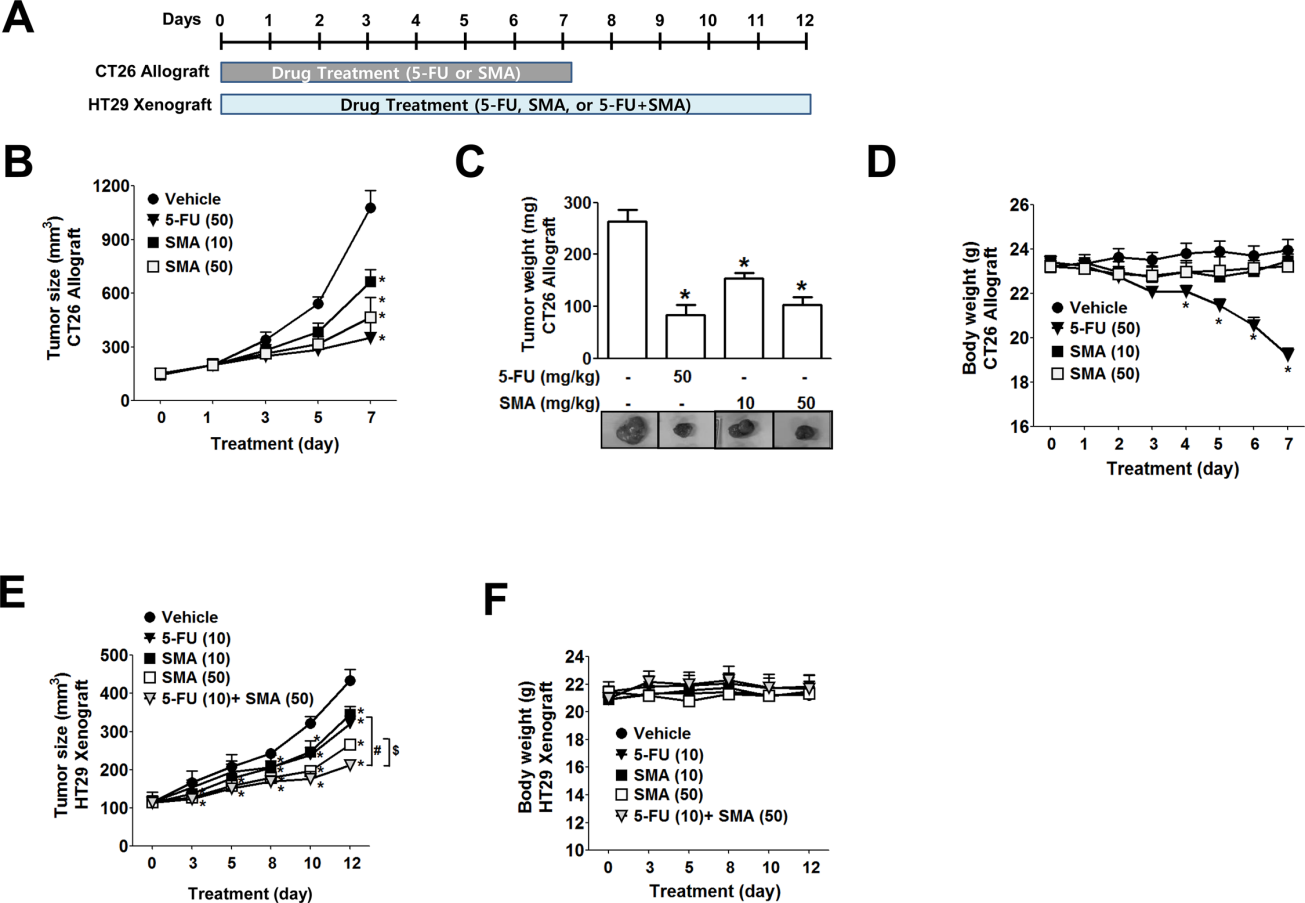
Suppression of colon tumor growth by SMA in murine syngenic and xenograft tumor models. CT26 murine or HT29 human colon cancer cells were subcutaneously injected into BALB/c mice or BALB/c nude mice, respectively. When tumors reached 120 mm^3^, SMA were administered intraperitoneally, and 5-FU was used as a positive control. (A) Schematic representation of drug treatment in CT26 allograft and HT29 xenograft tumor models. (B-D) Tumor growth in CT26 syngenic model as measured by tumor size (B) and excised tumor weights (C) along with body weight reductions (D). (E-F) In the HT29 xenograft tumor model, SMA dose-dependently inhibited tumor growth (E) without reducing body weight (F). ^*^ *P*<0.05 vs. the vehicle-treated controls. ^*^ *P*<0.05 vs. vehicle-treated controls. ^#^ *P*<0.05 vs. 5-FU alone-treated animals. ^$^ *P*<0.05 vs. SMA alone-treated animals. In all experiments, six mice per group were used.

To investigate the *in vivo* antitumor effect of SMA and its potential use in combination chemotherapy, we used a CT26 syngenic tumor model and a HT29 xenograft model in mice and compared the effects of SMA with the effects of 5-FU, which is commonly used to treat solid tumors, including colon cancer (drug treatment scheme is shown in Fig 4A). In the CT26 syngenic tumor model, SMA dose-dependently suppressed tumor growth in a manner similar to 5-FU (Fig 4B and 4C). However, 5-FU at 50 mg/kg showed obvious toxic effects, such as hair loss and significant body weight reduction at the fourth day of treatment (Fig 4D). Unlike 5-FU, SMA at the same dose did not produce such changes. Similarly, in the HT29 xenograft tumor model, SMA was found to have a dose-dependent anti-tumor effect (Fig 4E). In addition, when SMA (50 mg/kg) was administered with low dose 5-FU (10 mg/kg), the effects were greater than those of 5-FU (10 mg/kg) or SMA (50 mg/kg) alone (Fig 4D), and SMA did not reduce body weight (Fig 4F).

### Anti-tumor effect of SMA alone and in combination with cisplatin in the A549 lung cancer xenograft model

**Fig 5:**
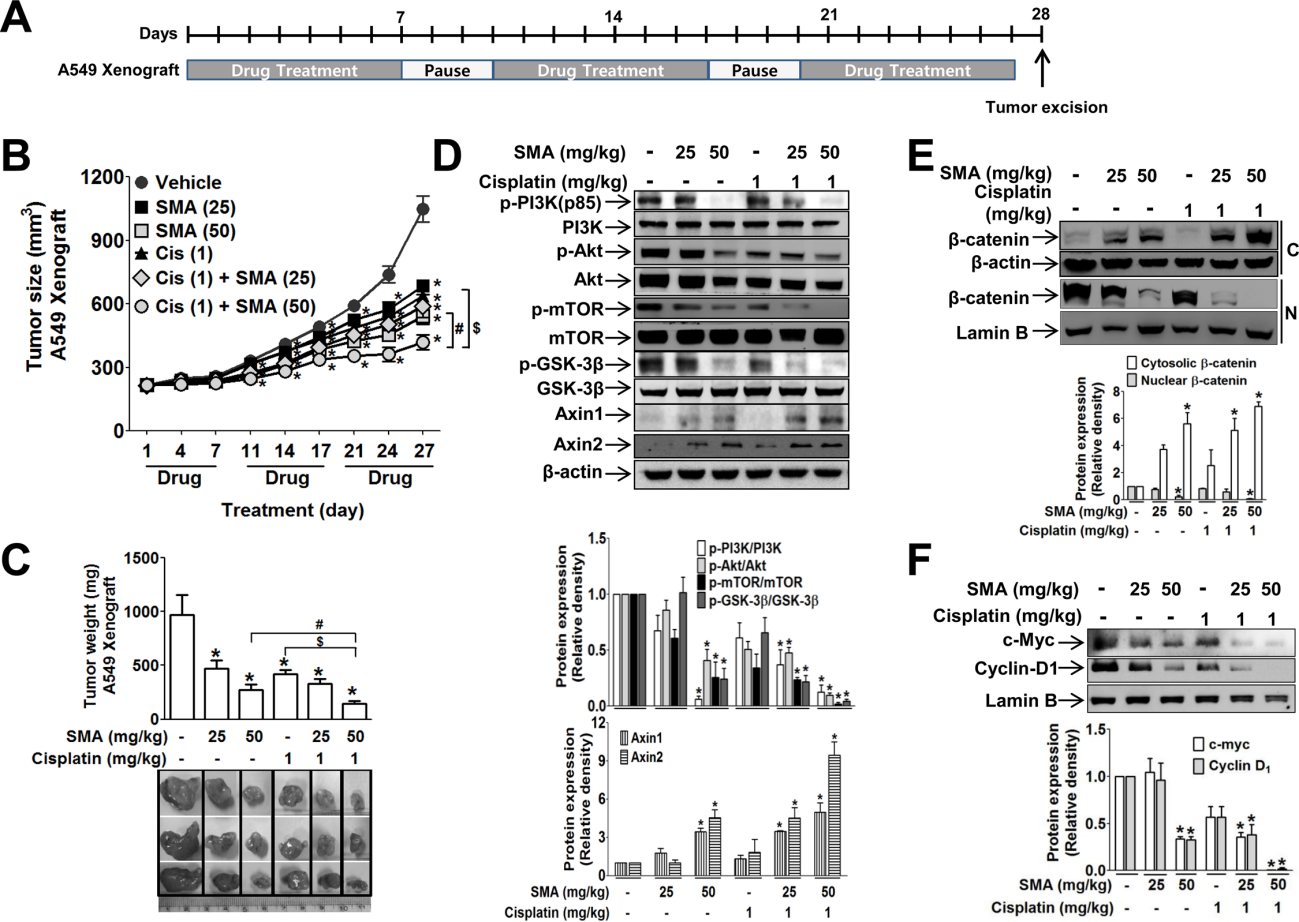
Anti-tumor effect of SMA alone and in combination with cisplatin in the A549 human non-small cell lung cancer xenograft model. (A) Tumor-bearing BALB/c nude mice were treated intraperitoneally with SMA, cisplatin, or both, for 7 consecutive days following a 3-day intermission as one cycle. Six mice per group were used. (B) Tumor growth was monitored by measuring tumor size. (C) Twenty eight days after commencing three cycles of treatment, tumor tissues were isolated and the tumor weights were measured. (D-F) Western blotting of tumor tissues on signaling molecule activation (D), nuclear localization of β-catenin (E), and protein expression of target genes (F). C and N in (E) represent cytosol and nucleus, respectively. ^*^ *P*<0.05 vs. vehicle-treated controls. ^#^ *P*<0.05 vs. cisplatin alone-treated animals. ^$^ *P*<0.05 vs. SMA alone-treated animals.

Next, we examined whether SMA could exert anti-proliferative activity in A549 human non-small cell lung cancer cells (NSCLC), which possess mutant APC and wild type β-catenin similar to HT29 colon cancer cells. Treatment with SMA inhibited A549 human lung cancer cell proliferation (Fig 1), and the IC_50_ value of SMA against serum- or GSK3β inhibitor-induced cancer cell proliferation was the lowest in A549 cells (Table 1). The anti-tumor effect and action mechanism of SMA were further examined in a mouse tumor model with a xenograft of A549 cells (drug treatment scheme is shown in Fig 5A). As shown in Fig 5B, SMA significantly suppressed tumor growth in this model. Because cisplatin is often the first treatment option for monotherapy or for combination chemotherapy in patients with advanced NSCLC, we also compared the effect of SMA with cisplatin. In the A549 xenograft model, 50 mg/kg SMA had an anti-tumor effect similar to 1 mg/kg cisplatin. Furthermore, combined therapy with cisplatin (1 mg/kg) and SMA (50 mg/kg) had a much stronger anti-tumor effect than cisplatin or SMA alone (Fig 5B and 5C). Western blot analyses of tumor tissues showed SMA significantly suppressed the phosphorylation of PI3K (p85), Akt, and GSK3β (Fig 5D). In contrast, axin levels were increased by SMA. Cisplatin treatment induced a similar but less potent effect on the phosphorylation of the signaling proteins. SMA in a combination regimen with cisplatin induced strong inhibitory effects on the signaling molecules. Nuclear β-catenin levels in tumor tissues were not inhibited by cisplatin alone, but were dramatically suppressed by SMA and synergistically by SMA plus cisplatin (Fig 5E). Regarding c-Myc and cyclin D1 levels, although cisplatin suppressed their nuclear levels, treatment with SMA plus cisplatin had a much stronger effect (Fig 5F).

## Discussion

In the present study, we demonstrate for the first time that the anti-cancer effects of SMA *in vitro* and *in vivo* are mediated through increased activity of the β-catenin destruction complex. The increased activity of the β-catenin destruction complex induced by SMA was supported by an increase in axin levels as well as suppression of tankyrase activity and increased GSK3β activity resulting from suppression of PI3K/Akt activity. The increased axin levels and GSK3β activity led to the phosphorylation and subsequent degradation of β-catenin in SMA-treated cancer cells (HT29) as well as tumor tissues (A549 xenograft tumor tissues). Together with the results of the previous studies [25,26], our findings suggest that SMA is a possible candidate as a multi-target anti-cancer agent that could be applied in combination therapy with other anti-cancer agents for synergistic effects.

Because of the important roles played by the oncogenic mutation of *PIK3CA* in cancer cell survival, proliferation, metabolism, and motility [29], PI3K is considered a molecular target for anti-cancer drug discovery [30]. Similarly, dysregulation of the Wnt/β-catenin pathway resulting in abnormal accumulation of β-catenin is associated with the pathogeneses and poor prognosis of both colon cancer and NSCLC [31]. Moreover, anti-cancer drug resistance in NSCLC is related to abnormal β-catenin [32]. Therefore, abnormal β-catenin signaling is also considered a target for cancer chemotherapy [33]. However, unlike PI3K/Akt inhibition by chemical compounds, direct inhibition of β-catenin signaling is difficult because β-catenin is regulated via many protein-protein interactions through the β-catenin destruction complex. The core components of the β-catenin destruction complex, which include APC, axin, CK1, and GSK3β, interact at extended protein-protein interfaces and are not considered viable molecular targets for drug development purposes. However, non-core tankyrase, an enzyme that causes degradation of axin which regulates GSK3β activity, has been viewed as a molecular target for anti-cancer drug development [17]. Suppression of HT29 colon cancer cells that harbor mutations in both the PI3K/Akt and Wnt/β-catenin pathways [34–36], requires dual-inhibitors for both pathways. Our present study showed that SMA fulfils anti-cancer agent criteria for inhibiting genetically heterogeneous cancers by inhibiting both PI3K/Akt and tankyrase, which is a regulatory component of GSK3β activity. Despite its low potency compared to XAV939, SMA showed a similar inhibitory effect on β-catenin-dependent TCF/LEF1 transcriptional activity. The results appear to be due to the dual inhibitory effects of SMA on PI3K/Akt and tankyrase, which results in a high level of GSK3β activation and nuclear localization of β-catenin. Furthermore, the present study also showed that A549 cells, which harbor mutant APC and wild-type β-catenin similar to HT29 cells, were more sensitive to SMA than HT29 cells. A549 cells express high levels of nuclear β-catenin, and this expression of nuclear β-catenin is reported to be strongly associated with a poor prognosis [37]. The differential effects of SMA on HT29 and A549 cells further support that SMA exerts a strong inhibitory action in nuclear β-catenin-dependent TCF/LEF1 transcriptional activity. However, there still is a possibility that SMA may directly inhibit nuclear translocation of β-catenin or binding of β-catenin to the TCF/LEF1 complex.

Our *in vivo* animal studies on CT26 syngenic and HT29 xenograft mouse models showed that the anti-tumor effect of SMA was dose-dependent and close to effect generated by 5-FU, which is the most commonly used cytotoxic anti-cancer drugs. However, 5-FU at 50 mg/kg daily causes significant side effects, such as hair loss and body weight reduction, whereas SMA did not cause such changes. Furthermore, we found combination treatment with 5-FU and SMA resulted in more potent anti-tumor activity than either drug alone without changing body weights, which suggests SMA could be used as an adjuvant therapeutic agent for CRC in combination with standard chemotherapy regimens. Cisplatin, which is most often used to treat NSCLC, has a number of associated side effects that limit its use. In particular, cisplatin nephrotoxicity, which is a major concern, is dose-related, and dose reductions are instituted when renal function is reduced. Our results show that SMA in combination with cisplatin synergistically increased anti-tumor activity in mice xenografted with A549 NSCLC without significant alteration in body weight (S1 Fig), which suggests SMA may be a valuable adjuvant therapeutic agent in combination with lower cisplatin doses. Additionally, similar to a previous report that silencing of β-catenin expression with siRNA inhibited drug resistance in A549 NSCLC [32], our present study showed a strong inhibitory action in nuclear β-catenin-dependent TCF/LEF1 transcriptional activity by SMA, also suggesting that SMA may be applied for cisplatin-resistant lung cancer. Our results also provide a basis for treating cancer stem cells that possess unlimited proliferative and self-renewal abilities and are resistant to chemotherapy [38,39]. Wnt/β-catenin signaling is critically involved in the maintenance of cancer stem cells in a variety of cancers including colon [40] and lung [32] cancers. Our current results showing SMA exerted a dose-dependent and strong inhibitory activity against β-catenin-dependent TCF/LEF1 transcriptional activity suggest SMA as an inhibitor against cancer stem cells.

In conclusion, our findings suggest SMA could function as a potential monotherapy or in combination with cytotoxic chemotherapeutic drugs, such as 5-FU or cisplatin. The molecular basis for the anti-tumor activity of SMA involves increased activity of the β-catenin destruction complex through the inhibition of tankyrase and PI3K/Akt pathway.

## Supporting Information

S1 Fig Supporting figure. (TIF)
