# Supplementary figures and images for: The Anti-Tumor Activity of Succinyl Macrolactin A Is Mediated through the β-Catenin Destruction Complex via the Suppression of Tankyrase and PI3K/Akt
Source: PLoS One. 2015 Nov 6;10(11):e0141753. doi: 10.1371/journal.pone.0141753 (PMC4636297; doi:10.1371/journal.pone.0141753)

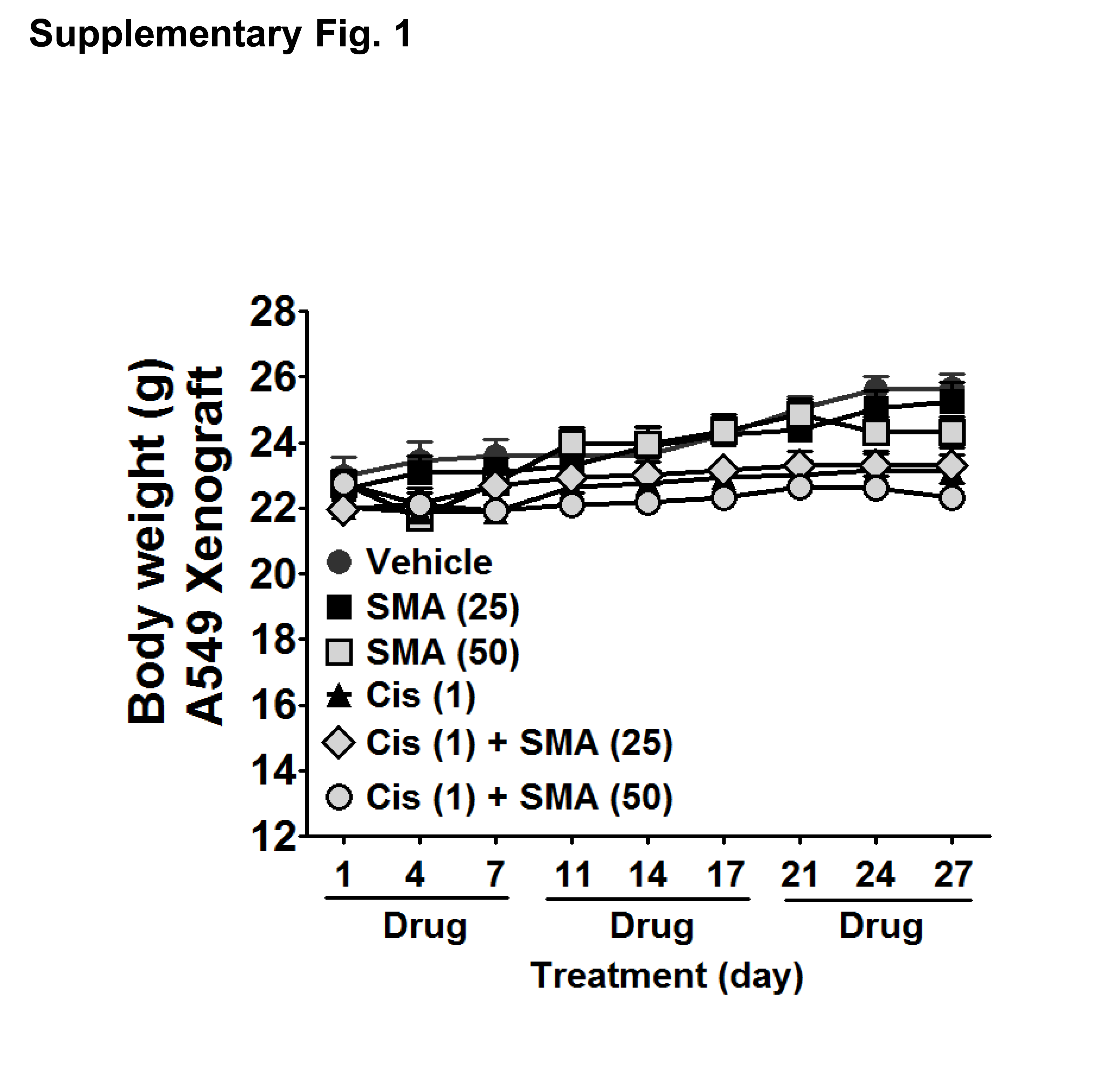

Supplement: S1 Fig — (TIF) [file pone.0141753.s001.tif]
